# Diesel exhaust alters the response of cultured primary bronchial epithelial cells from patients with chronic obstructive pulmonary disease (COPD) to non-typeable *Haemophilus influenzae*

**DOI:** 10.1186/s12931-017-0510-4

**Published:** 2017-01-28

**Authors:** Maria C. Zarcone, Annemarie van Schadewijk, Evert Duistermaat, Pieter S. Hiemstra, Ingeborg M. Kooter

**Affiliations:** 1 Department of Pulmonology, Leiden University Medical Center, Albinusdreef 2, 2333 ZA Leiden, The Netherlands; 2 Triskelion BV, Zeist, The Netherlands; 3 Netherlands Organization for Applied Scientific Research, Utrecht, The Netherlands

**Keywords:** Airway epithelial cells, Diesel exhaust, Air pollution, COPD, Non-typeable *Haemophilus influenzae*, Integrated stress response, Antimicrobial response, Antimicrobial peptides

## Abstract

**Background:**

Exacerbations constitute a major cause of morbidity and mortality in patients suffering from chronic obstructive pulmonary disease (COPD). Both bacterial infections, such as those with non-typeable *Haemophilus influenzae* (NTHi), and exposures to diesel engine emissions are known to contribute to exacerbations in COPD patients. However, the effect of diesel exhaust (DE) exposure on the epithelial response to microbial stimulation is incompletely understood, and possible differences in the response to DE of epithelial cells from COPD patients and controls have not been studied.

**Methods:**

Primary bronchial epithelial cells (PBEC) were obtained from age-matched COPD patients (*n* = 7) and controls (*n* = 5). PBEC were cultured at the air-liquid interface (ALI) to achieve mucociliary differentiation. ALI-PBECs were apically exposed for 1 h to a stream of freshly generated whole DE or air. Exposure was followed by 3 h incubation in presence or absence of UV-inactivated NTHi before analysis of epithelial gene expression.

**Results:**

DE alone induced an increase in markers of oxidative stress (HMOX1, 50–100-fold) and of the integrated stress response (CHOP, 1.5–2-fold and GADD34, 1.5-fold) in cells from both COPD patients and controls. Exposure of COPD cultures to DE followed by NTHi caused an additive increase in GADD34 expression (up to 3-fold). Importantly, DE caused an inhibition of the NTHi-induced expression of the antimicrobial peptide S100A7, and of the chaperone protein *HSP5A*/BiP.

**Conclusions:**

Our findings show that DE exposure of differentiated primary airway epithelial cells causes activation of the gene expression of HMOX1 and markers of integrated stress response to a similar extent in cells from COPD donors and controls. Furthermore, DE further increased the NTHi-induced expression of GADD34, indicating a possible enhancement of the integrated stress response. DE reduced the NTHi-induced expression of S100A7. These data suggest that DE exposure may cause adverse health effects in part by decreasing host defense against infection and by modulating stress responses.

**Electronic supplementary material:**

The online version of this article (doi:10.1186/s12931-017-0510-4) contains supplementary material, which is available to authorized users.

## Background

Exposure to particulate air pollution is associated with a range of adverse health effects, including respiratory infections [1]. Diesel exhaust (DE) constitutes the major source of traffic-related air pollution in the most densely populated areas [2]. Exposures to traffic pollution have been associated with development of lung disease [3–5] and an increased risk for patients with pre-existing lung disease for development of symptoms [2, 6]. Exposure to particulate air pollution is also associated with exacerbations in patients with chronic obstructive pulmonary disease (COPD), linking COPD exacerbations to episodes of increased (traffic-related) air pollution [7–9]. This may be the result of higher susceptibility of COPD patients to the adverse effects of DE. Most COPD exacerbations are associated with bacterial and/or viral respiratory infections, as illustrated by the presence of bacteria such as non-typeable *Haemophilus influenzae* (NTHi) in the lower respiratory tract of 50% of patients during exacerbations [10]. However, since urban air pollution is a mixture of DE and other air pollutants and several variables can influence individual exposures, a direct link between traffic-related air pollution and infections has not been established based on observational studies [2]. Experimental studies have clearly shown that diesel particles impair host defense by suppressing e.g. macrophage and epithelial cell function [11–13]. So far the effect of whole DE (a complex mixture of both particles and gaseous component) on host defense function of cultured primary human airway epithelial cells has not been studied.

The airway epithelium constitutes the first barrier for inhaled toxic compounds such as DE and respiratory pathogens [14, 15]. The airway epithelium of COPD patients is characterized by an increased susceptibility to infections, reduced antimicrobial response and increased oxidative stress and integrated stress response [15]. *In vitro* cultures of epithelial cells from COPD patients have revealed a partial persistence of the COPD phenotype in culture [16, 17]. This is important since the airway epithelium can exert an active function in the innate immune responses by releasing antimicrobial peptides and proteins (AMPs), such as human beta-defensin (hBD)-2 (encoded by the gene *DEFB4A*), S100 calcium binding protein (S100A7), lipocalin-2 (LCN2) and secretory leukocyte protease inhibitor (SLPI) [14, 15]. Previous studies showed that treatment of A549 lung epithelial tumor cells with resuspended air pollution particles reduced hBD-2 [11], while coal fly ash interfered with *Pseudomonas aeruginosa* bacterial killing by primary bronchial epithelial cells *in vitro* and in a mouse model *in vivo* [18].

We and others have used whole DE (instead of resuspended particles) to investigate effects of diesel on human cells, and demonstrated that it increases markers of the oxidative stress response, such as heme oxygenase 1 protein (HO-1, encoded by *HMOX1* mRNA) in A549 cells and primary bronchial epithelial cells [19–21], as well as production of pro-inflammatory mediators, including CXCL8 [22]. We also showed that whole DE causes activation of the integrated stress response (ISR) in human bronchial epithelial cells [21]. A key and early event in activation of the ISR is the phosphorylation of the initiation factor of protein translation eIF2α. This phosphorylation can be mediated by four different kinases, PERK (protein kinase R (PKR)-like endoplasmic reticulum kinase), HRI (heme-regulated eIF2α kinase), GCN2 (general control nonderepressible kinase 2) and PKR (protein kinase R), which are activated by specific stimuli. Phosphorylation of eIF2α results in inhibition of protein synthesis, and preferential transcription of the transcriptional factor ATF4 which induces expression of CHOP and GADD34 [23]. In addition, cell injury induced by oxidative stress may result in an unfolded protein response (UPR), in which PERK-mediated eIF2α phosphorylation occurs simultaneously with the activation of IRE1α, generating spliced XBP1, and ATF6 (both inducing expression of chaperones such as BiP [23, 24]).

Since activation of the airway epithelium by respiratory pathogens such as NTHi is a central event during COPD exacerbations, we focused on the ability of DE to modulate this activation. We hypothesized that the effect of diesel differs between epithelial cells from COPD patients and controls and that whole DE impairs production of AMPs by primary differentiated airway epithelial cells. Primary bronchial epithelial cells (PBECs) from COPD patients and controls were cultured at the air-liquid interface (ALI) to achieve mucociliary differentiation. To adequately mimic the *in vivo* exposure of epithelial cells to DE, exposures were performed with DE produced by a non-road mobile machinery stage IIIb [21], before addition of UV-inactivated non-typeable *Haemophilus influenzae* (NTHi).

## Methods

### Bronchial epithelial cell culture and donor characterization

Cells were obtained from macroscopically normal and tumor-free lung tissue from 5 non-COPD and 7 COPD donors undergoing resection surgery for lung cancer at the Leiden University Medical Center. Patient groups were matched for age. Disease status of COPD donors (two GOLD III, three GOLD II and two GOLD I) was based on lung function according to the Global Initiative for Chronic Obstructive Lung Disease (GOLD) classification [25]. Mean FEV_1_ % predicted and FEV_1_/FVC were significantly lower in COPD patients compared to controls. Two COPD donors were ex-smokers (3 and 6 years) and three were current smokers. In the non-COPD group one patient never smoked, three were ex-smokers and one was a current smoker (Table 1). No information on smoking history was available for two COPD donors.

**Table 1:** Donor characterization

	COPD	SD	non-COPD	SD
Gender	5/2 (M/F)	-	4/1 (M/F)	-
AGE, years	62.57	7.41	66.60	2.70
BMI (%)	24.55	2.13	26.70	1.09
FEV_1_ (%predicted)	67.46	22.84	94.82	24.09
FEV_1_/FVC (%)^a^	54.83	9.20	75.51	4.49
Smoking history (never smoker/ex-/current smoker)	-/2/3^b^	-	1/3/1	-

^a^ *p* < 0.01 vs non-COPD subjects by Mann–Whitney *t*-test

^b^No information on smoking history available for two COPD donors

Primary bronchial epithelial cells (PBECs) obtained from bronchial ring tissue were first expanded submerged in keratinocyte serum free medium (KSFM, Life technologies) supplemented with penicillin (Lonza, Verviers, Belgium), streptomycin (Lonza), epithelial growth factor (EGF, Life technologies), bovine pituitary extract (BPE, Gibco) isoproterenol (Sigma-Aldrich, St. Louis, USA), and ciprofloxacin (Fresenius Kabi, Schelle, Belgium) as previously described [21]. Then cells were seeded onto 12 well-plate Transwell inserts (Corning Costar Corporation, Cambridge, MA) and cultured in BEBM in a 1:1 mix with DMEM (Lonza) supplemented with BEGM SingleQuot (Lonza), penicillin/streptomycin (Lonza), BSA (1 mg/ml, Sigma-Aldrich) and additional retinoic acid (15 ng/ml, Lonza). After reaching confluence, apical medium was removed and cells were cultured at the air-liquid interface (ALI) for two weeks to allow mucociliairy differentiation, as demonstrated by the presence of ciliated and mucus-producing cells [26].

### Whole diesel exposure system

Exposures of ALI-PBECs to air or diesel exhaust (DE) were performed in Vitrocell® units (Waldkirch, Germany) in triplicate as previously described [21]. Emissions were produced by a diesel engine comparable to a stage IIIb non-road diesel engine operating at a steady load. DE was diluted immediately 9-times with humidified air to generate a mixture that was defined as DE, and that in a previous study was found to induce cellular responses in ALI-PBEC [21]. Particle matter (PM) concentrations in the 9-times diluted DE were quantified by TSI scanning mobility particle sizer (SMPS; model 3936 L22 TSI Incorporated, Shoreview, MN, USA). PM concentration was assessed during 1 h exposure to DE by SMPS, and shown to be 1.51 ± 0.12 mg/m^3^. The dose delivered to the cells (delivered dose, DD) was calculated to be 0.40 μg/cm^2^ based on flow velocity, PM concentration, time of exposure and transwell surface area, as previously described [21]. The deposited dose (*dd*) on inserts was calculated to be 6.86 ng/cm^2^ based on a calculated deposition efficiency of 1.7% of the delivered dose as described [21]. During each exposure session, temperature (average of twelve exposures: 23.64 °C with 0.50 °C as standard deviation), relative humidity (63.57 ± 1.57%), carbon dioxide (0.46 ± 0.02%) and oxygen content (19.98 ± 0.04%) were monitored and maintained constant. Based on estimation of the delivered doses, we previously calculated that 1 h exposure used in our *in vitro* exposure system corresponded to 2.25 h exposure *in vivo* to a relatively high level of pollution (50 μg/m^3^) [26, 27].

### UV-inactivated non-typeable *Haemophilus influenzae*

Non-typeable *Haemophilus influenzae* (NTHi) strain D1 [28] was cultured as previously described [29]. Briefly, bacteria were grown on chocolate agar plates (bioMérieux, Zaltbommel, The Netherlands) and one single colony was transferred into 10 ml of Tryptic Soy Broth (TSB) plus hemin (factor X) and nicotinamide adenine dinucleotide (NAD or factor V; TSB plus factor X and V, Mediaproducts BV, Groningen, the Netherlands) and incubated while shaking overnight at 37 ° C. Two ml of this overnight culture was inoculated in a fresh tube with 10 ml of TSB plus factor X and V for 4 h while shaking at 37 °C to obtain a log-phase culture. NTHi bacteria were harvested by centrifugation for 10 min at 1840 g, re-suspended in PBS and quantified at OD_600_ nm. Next bacteria were diluted in PBS to a concentration of 1*10^9^ CFU/ml, and inactivated by exposure to UV-light for 2 h.

### Exposure to whole diesel exhaust and other stimuli

Cells from 5 non-COPD and 7 COPD donors were exposed in triplicate to air or high DE (9-fold diluted, DD 0.40 μg/cm^2^) as previously described [21]. Briefly, at 24 h before exposure the apical side of ALI-PBECs was washed with 100 μl of PBS to remove mucus and cell debris. For each condition, three inserts per donor were exposed to DE or air within Vitrocell® exposure units with 3 ml of medium in the basal compartment. After 1 h exposure, the Transwell inserts (Corning Costar Corporation, Cambridge, MA) were transferred into 12 well-plates with fresh media.

After 1 h exposure to air or high DE, 100 μl of PBS or 100 μl of 1 × 10^9^ CFU/ml of UV-inactivated NTHi in PBS was added to the apical side of the cultures and incubated for 3 h. In addition, inserts from the same donors that were not exposed to air or DE in the exposure unit, were incubated in duplicate with fresh media as untreated controls or treated with TGFβ (20 ng/ml; R&D system), TNFα (20 ng/ml; Peprotech), tunicamycin (Tm; 5 μg/ml, Sigma) that were added to the basolateral compartment, or with NTHi added to the apical surface of the cells, as positive controls for oxidative stress response, inflammatory response, unfolded protein response and antimicrobial response respectively. All controls were incubated with 100 μl of PBS apically.

### Transepithelial electrical resistance (TEER) and LDH release

Transepithelial electrical resistance was measured using an electrometer EVOM2 (World Precision Instruments, Sarasota, FL). Ohm values were subsequently multiplied by the surface of the Transwell inserts (1.12 cm^2^) to obtain the unit area resistance which was expressed as Ohm*cm^2^. Cytotoxic effects were investigated using the LDH detection Kit (LDH detection Kit, Roche, ver. 10) by assessment of LDH in basal media and apical washes, and expressed as % release of the positive controls treated with 0.01% (v/v) TRITON-X100 (Sigma).

### Quantitative real-time PCR

Total RNA was extracted using the Maxwell® 16 simplyRNA Tissue Kit (Promega, Leiden, NL) as described [21]. RNA samples were then converted to cDNA by adding MML-V enzyme (Promega), oligo(dT) primers and RNAsin (Promega). Quantitiave PCR reactions were performed using the CFX-384 RT-PCR detection system (Bio-Rad Laboratories, Veenendaal, The Netherlands) and iQSybr green Supermix (Biorad). Gene expression was assessed with the standard curve method (Bio-Rad CFX manager 3.0 software, Bio-Rad) for markers of oxidative stress (*HMOX1*), unfolded protein response (*DDIT3/CHOP*, *PPP1R15A/GADD34*, *HSPA5/BiP* and *spliced XBP1*), inflammation (*CXCL8*) and antimicrobial response (*DEFB4A*/hBD2 and *S100A7*). Arbitrary gene expression levels were normalized using expression of the reference genes *ATP5b* and *RPL13a*, which were selected using the GeNorm method [30]. All primers sequences, temperatures and gene ID are indicated in Table 2.

**Table 2:** Primer sequences for quantitative qPCR

Gene	Tm (°C)	Forward Sequence / Reverse sequence	GeneBank accession no. or reference
*ATP5B*	63°	TCACCCAGGCTGGTTCAGA	NM_001686
		AGTGGCCAGGGTAGGCTGAT	
*RPL13A*	63°	AAGGTGGTGGTCGTACGCTGTG	NM_012423
		CGGGAAGGGTTGGTGTTCATCC	
*MUC5AC*	65°	CCTTCGACGGACAGAGCTAC	[42]
		TCTCGGTGACAACACGAAAG	
*FOXJ1*	65°	GGAGGGGACGTAAATCCCTA	[29]
		TTGGTCCCAGTAGTTCCAGC	
*HMOX1*	63°	AACCCTGAACAACGTAGTCTGCGA	NM_002133
		ATGGTCAACAGCGTGGACACAAA	
*HSPA5*/BiP	62°	CGAGGAGGAGGACAAGAAGG	NM_001025433
		CACCTTGAACGGCAAGAACT	
*DDIT3/CHOP*	62°	GCACCTCCCAGAGCCCTCACTCTCC	NM_001195053.1
		GTCTACTCCAAGCCTTCCCCCTGCG	
*PPP1R15A/GADD34*	62°	ATGTATGGTGAGCGAGAGGC	[43]
		GCAGTGTCCTTATCAGAAGGC	
*splXBP1*	62°	TGCTGAGTCCGCAGCAGGTG	[44]
		GCTGGCAGGCTCTGGGGAAG	
*CXCL8*	59°	CTG GAC CCC AAG GAA AAC	NM_000584
		TGG CAA CCC TAC AAC AGA C	
*DEFB4A*/hBD2	62°	ATCAGCCATGAGGGTCTTG	NM_004942
		GCAGCATTTTGTTCCAGG	
*S100A7*	60°	ACGTGATGACAAGATTGACAAGC	NM_002963.3
		GCGAGGTAATTTGTGCCCTTT	

### Statistical analysis

For each donor, means were calculated from the triplicate experimental conditions. Within non-COPD and COPD groups, the effects of exposure conditions were compared to relative controls using a two-tailed One-Way ANOVA with Bonferroni’s correction to take repeated measures into account. Differences in response to DE or DE and NTHi between non-COPD and COPD donors were compared with a nonparametric *t*-test for independent samples (Mann–Whitney test). Differences were considered statistically significant at *p* < 0.05.

## Results

### Study design and epithelial cell characteristics

Based on previous findings [21], we selected exposure for 1 h to DE to mimic a short, transient exposure to DE. In pilot experiments, 3 h post-exposure incubation was found to be optimal to study DE-induced expression of the antioxidant response, ISR, and NTHi-induced inflammatory and antimicrobial responses at the mRNA level. To compare epithelial differentiation in cultures from COPD and non-COPD donors, gene expression of differentiation marker was analyzed. Expression of *MUC5AC* (oligomeric mucus/gel-forming, marker for mucus producing cells) was higher in COPD cultures, but this difference did not reach statistical significance (*p* = 0.073). *FOXJ1* (forkhead box J1, marker for ciliated cells) appeared lower in COPD, but this difference was not significant (Additional file 1: Figure S1A and S1B). While no disease-related differences were observed in expression of the other markers studied in untreated controls (data not shown).

### Epithelial barrier and cytotoxicity

**Fig. 1:**
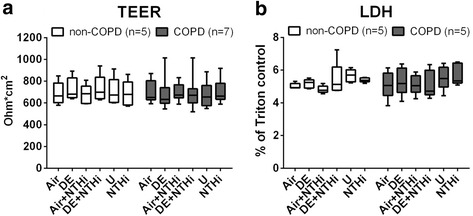
Epithelial barrier and cytotoxicity. Cells from 5 non-COPD and 7 COPD donors were exposed for 1 h to air (Air) or diesel exhaust (DE) and then incubated for 3 h with or without UV-inactivated NTHi that was added to the apical side (Air + NTHi or DE + NTHi). As controls, inserts not placed in the exposure units were also incubated for 3 h as untreated (U) or with NTHi alone (NTHi). TEER values (trans-electrical epithelial resistance) were measured and expressed as Ohm*cm^2^ **a**, while LDH release in the apical and basal compartment was quantified and expressed as % of the positive control (0.01% TRITON-X 100) **b**. Data are shown as boxes with median, with the whisker indicating the minimum and the maximum values detected. No significant differences were observed (two-tailed One-Way ANOVA with Bonferroni’s correction)

Effects of DE exposure followed by exposure to UV-inactivated NTHi on epithelial barrier activity (TEER) and cytotoxicity (LDH release) were assessed in cells from 5 non-COPD and 7 COPD donors. No effect of these treatments on TEER and cytotoxicity was observed in both groups of patients (Fig. 1a and b).

### Gene expression of heme oxygenase-1 and of genes involved in the integrated stress response (ISR)

**Fig. 2:**
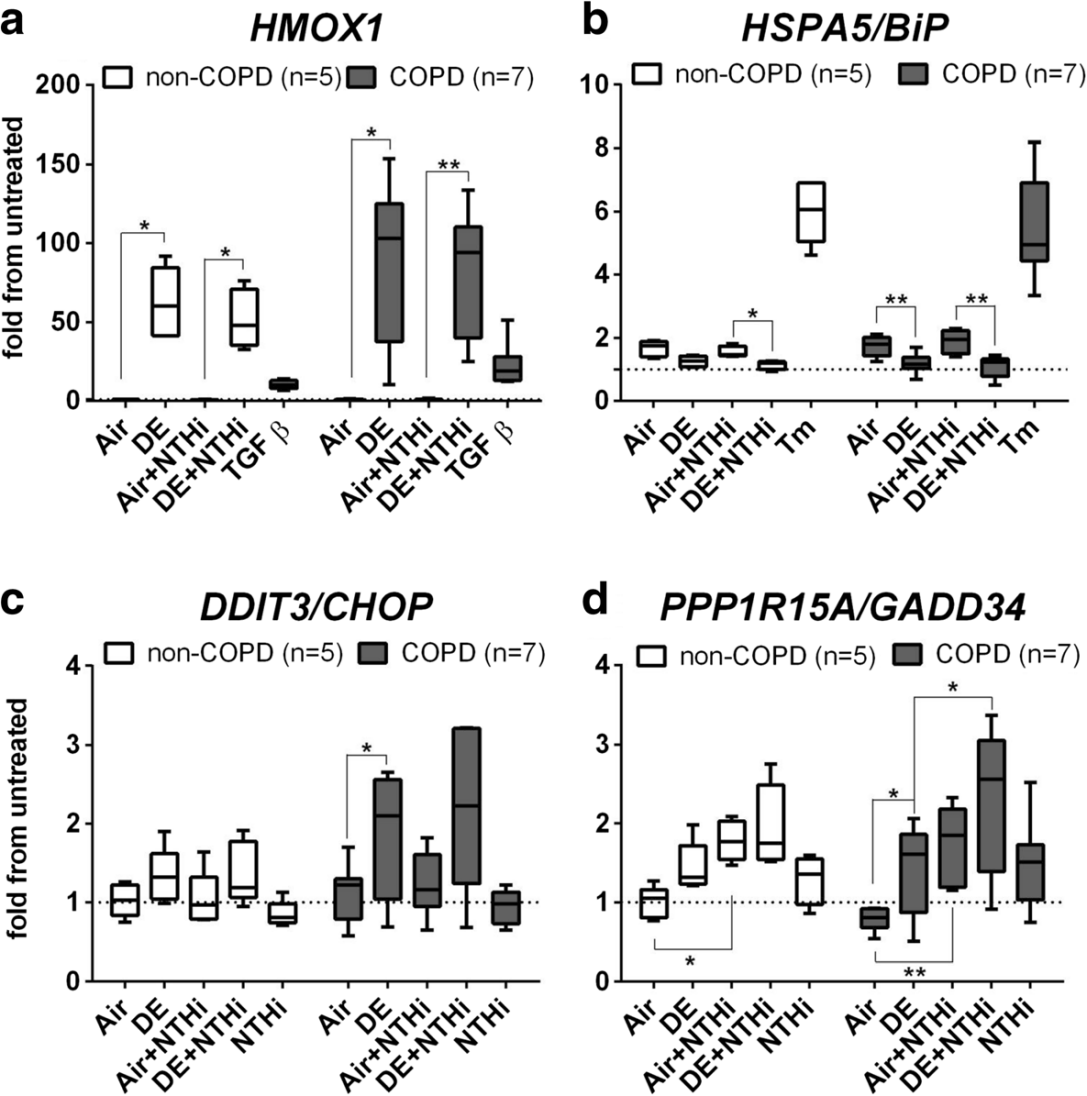
Gene expression of heme oxygenase-1 and of genes involved in the integrated stress response (ISR). ALI-PBECs from COPD and control donors were exposed for 1 h to air (Air) or DE, followed by 3 h exposure to UV-inactivated NTHi (Air + NTHi or DE + NTHi). TGFβ (20 ng/ml) is shown as positive control for the oxidative stress response **a**, tunicamycin (20 ng/ml, Tm) for the BiP expression **b** and controls with NTHi alone (NTHi). Expression was assessed of *HMOX1* mRNA as marker of the oxidative stress response **a**, and of *HSPA5* **b**, *DDIT3/CHOP* **c** and *PPP1R15A/GADD34* **d** as markers of the unfolded protein response (UPR) to ER stress. Data are shown as boxes with median, with the whisker indicating the minimum and the maximum values detected. mRNA induction is expressed as fold from untreated control (indicated by a dashed line) after normalization on two reference genes. Statistical significance of differences is indicated as * *p* < 0.05, ** *p* < 0.01 and *** *p* < 0.001 and was assessed using a two-tailed One-Way ANOVA with Bonferroni’s correction

Cells from 5 non-COPD and 7 COPD donors were exposed to DE followed by exposure to NTHi, and analyzed for mRNA expression. In cells from both non-COPD and COPD donors, DE significantly increased *HMOX1* mRNA expression (marker for oxidative stress response) both in presence (*p = 0.0104 for non-COPD and *p* = 0.0128 for COPD donors) or absence of NTHi exposure (*p = 0.0101 and **p = 0.0068, Fig. 2a). DE did not increase expression of *HSPA5* (encoding BiP, a chaperone protein marker for the unfolded protein response [UPR] to endoplasmic reticulum stress; Fig. 2b) or *spliced XBP1* (data not shown), in contrast to tunicamycin (Tm; used as a positive control). DE significantly inhibited *HSPA5* expression in COPD donors (**p = 0.0045, Fig. 2b). *HSPA5* expression was also reduced by DE in cells treated with NTHi, compared to the air controls incubated with NTHi, with a significant reduction in both COPD and controls group (**p = 0.0014 and *p = 0.0230 respectively, Fig. 2b). Tunicamycin (Tm) caused a marked increase in *HSPA5/*BiP (Fig. 2b), *DDIT3/CHOP*, *PPP1R15A/GADD34* (Additional file 2: Figure S2A and S2B) and *spliced XBP1* (data not shown), indicating that all pathways of the UPR are activated in both COPD and non-COPD epithelial cells. DE exposure also increased expression of both markers of the ISR, both in presence and absence of NTHi: *DDIT3/CHOP* mRNA was increased in cells from both non-COPD and COPD donors after DE exposure; this DE-induced increase in *DDIT3/CHOP* only reached significance in cells from COPD patients (Fig. 2c; *p = 0.0493), whereas the further increase in presence of NTHi after DE exposure did not reach statistical significance. *PPP1R15A/GADD34* was also significantly increased after DE exposure in COPD donors (Fig. 2d; *p = 0.0265). Furthermore, the difference in expression of *PPP1R15A/GADD34* between cells exposed to DE with or without subsequent NTHi treatment was significant only in COPD donors (*p = 0.0182). Similarly, *PPP1R15A/GADD34* expression was significantly increased by NTHi alone in air-exposed cells (*p = 0.0132 in controls groups and **p = 0.0011 in COPD), whereas *DDIT3/CHOP* was not affected. Finally, NTHi alone also increased *PPP1R15A/GADD34* expression in cells not present in the exposure modules, but this increase did not reach statistical significance (data not shown).

### Expression of genes involved in the inflammatory and antimicrobial response

**Fig. 3:**
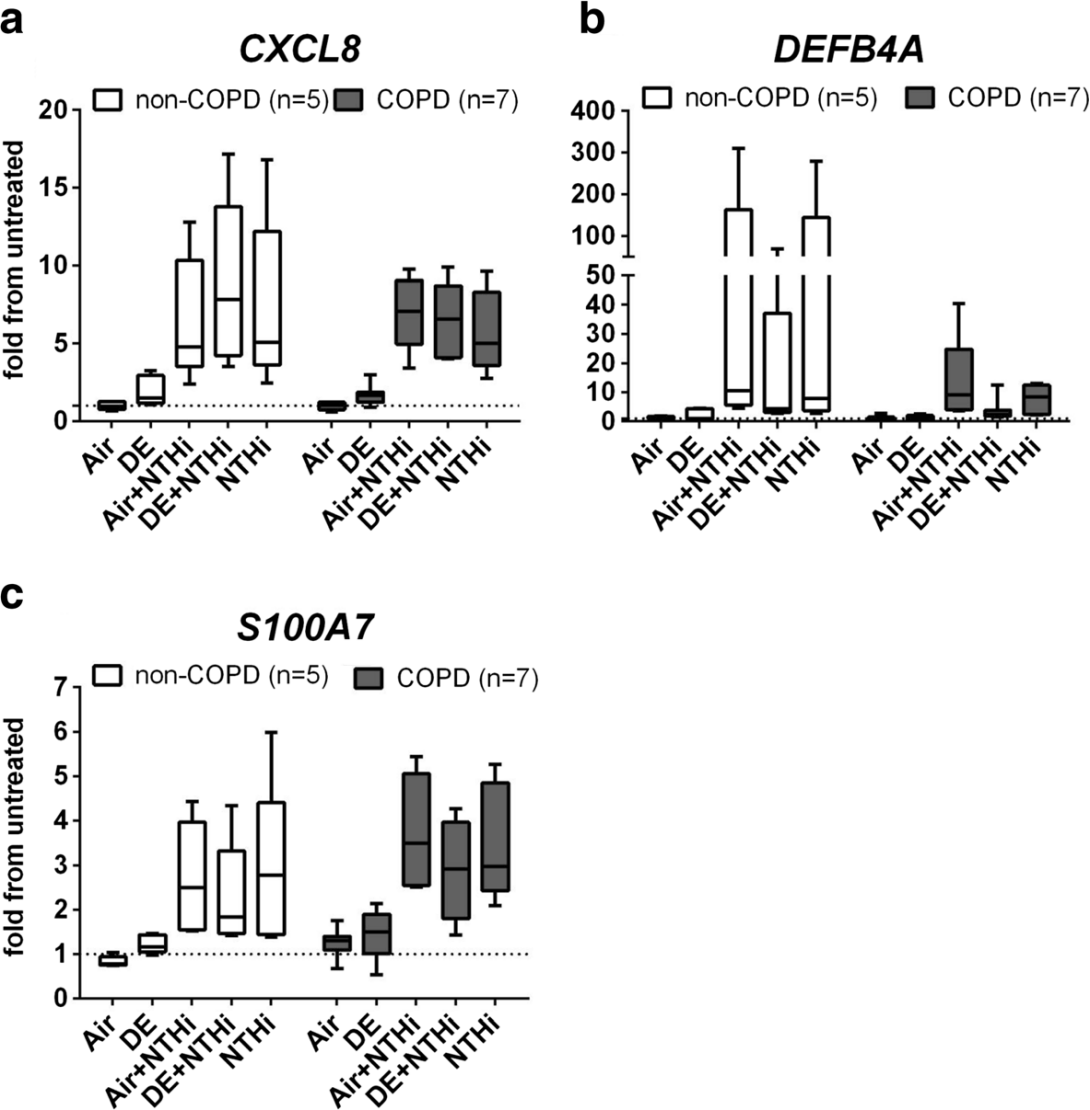
Expression of *CXCL8* and antimicrobial proteins. ALI-PBECs from COPD and control donors were exposed for 1 h to air (Air) or DE, followed by 3 h exposure to UV-inactivated NTHi (Air + NTHi or DE + NTHi); cultures not exposed in the exposure units but treated with NTHi alone (NTHi) are reported as positive controls. Analysis of gene expression of the neutrophil attracting chemokine *CXCL8* **a**, the antimicrobial peptide *DEFB4A* **b**, and *S100A7* **c** is shown. Data are shown as boxes with median, with the whisker indicating the minimum and the maximum values detected. mRNA induction is expressed as fold from the untreated control (indicated by a dashed line) after normalization on two reference genes. Statistical significance of differences is indicated as * *p* < 0.05, ** *p* < 0.01 and ***p < 0.001 and was assessed using a two-tailed One-Way ANOVA with Bonferroni’s correction. When combining the non-COPD and COPD groups, a statistically significant induction of *CXCL8* mRNA by DE was observed (*p* = 0.0064; data not shown in the figure) as well as a significant DE-mediated inhibition of the NTHi-induced *S100A7* mRNA (*p* = 0.0155; data not shown in the figure)

NTHi caused a marked and significant increase in *CXCL8* mRNA in all donors, whereas its induction following DE exposure alone was only modest and did not reach statistical significance (Fig. 3a). Only after combining the non-COPD and COPD groups, we observed a statistically significant induction of *CXCL8* mRNA (*p = 0.0064, not shown) by DE. NTHi also significantly increased expression of *S100A7* after combining the non-COPD and COPD groups (***p = 0.0002, not shown). Expression of both *DEFB4A*/hBD2 and *S100A7* was inhibited by prior DE exposure, without reaching statistical significance, due to the substantial inter-donor variation (Fig. 3b and c). Again, after combining the COPD and control group in the analysis, the DE-mediated inhibition of *S100A7* was found to reach significance (*p = 0.0155, not shown). Similar observations on responses of cells exposed to TNFα instead of NTHi were made (Additional file 3: Figure S3 and Additional file 4: Figure S4). Three hours incubation with UV-NTHi was not sufficient to increase *LCN2* or *SLPI* mRNA levels (data not shown).

## Discussion

In the present study, we showed that primary bronchial epithelial cells from COPD and non-COPD patients respond to diesel exhaust (DE) exposure by an increased expression of the oxidative stress response gene *HMOX1* and of *CXCL/IL-8*, and activation of the integrated stress response (ISR), while no effect on barrier function or cell death was found in both groups. Although there was a tendency for higher responses to DE in COPD patients, this did not reach statistical significance. Furthermore, NTHi significantly increased expression of *PPP1R15A/GADD34* mRNA in absence of activation of other investigated markers of the UPR or ISR, and this expression was further increased by prior DE exposure in COPD donors. In contrast, DE alone significantly reduced expression of the chaperone *HSPA5*/BiP in COPD donors. The DE-mediated *HSPA5*/BiP inhibition was significant in both groups in NTHi-treated cultures. The ability to mount an unfolded protein response (UPR) or ISR in response to tunicamycin did not differ between COPD and controls. Importantly, DE exposure appeared to inhibit the ability of NTHi to increase gene expression of the antimicrobial peptide *DEFB4A*/hBD-2 and S100A7, but this only reached statistical significance for S100A7 after combining the COPD and control group.

We previously showed that diesel induces transcription of heme-oxygenase-1 (HO-1) mRNA (*HMOX1)* [21]. Here we confirm this DE-mediated induction of *HMOX1* in primary cells using a shorter exposure time and therefore lower deposited dose of DE. At 3 h after exposure, *HMOX1* mRNA was increased in all cells exposed to DE (~50–100-fold), with a non-significant reduction in presence of NTHi in both donor groups. Several pathways are involved in induction of *HMOX1* mRNA, including redox sensitive activation of the transcription factor Nrf2 [31] which has been shown to be modulated by cigarette smoke [32, 33]. Recently, it has been suggested that ATF4 can act in concert with Nrf2 to induce *HMOX1* expression as a protective mechanism preventing apoptosis of tumor cells [34]. ATF4 is a key factor in the ISR and in PERK-mediated activation of the UPR. Here we showed that DE exposure increased *DDTI3/CHOP* and *PPP1R15A/GADD34* mRNA, which is indicative of ATF4 translation and action [23]. No cytotoxic effects were observed at 3 h incubation, and furthermore we previously showed absence of cytotoxicity at 24 h post-exposure using the same exposure conditions [21]. Whereas our data suggest involvement of ATF4, further studies are needed to clarify which transcriptional factors contribute to the induction of *HMOX1* mRNA after DE exposure and to understand its role in protection against DE.

*DDTI3/CHOP* and *PPP1R15A/GADD34* mRNA induction occurred without a concomitant increase of *HSPA*/BiP or spliced XBP1 [data not shown], markers for the other two UPR arms. This confirms our previous observation on a possible involvement of the ISR in the epithelial response to DE [21], using shorter exposure durations. For the first time, we demonstrated that NTHi causes a selective increase in GADD34, without activation of the other investigated markers of the ISR (CHOP) or UPR. Furthermore, we observed that this increase is enhanced by prior exposure to DE. In a previous study, we showed that *Pseudomonas aeruginosa* (PAO1) (also associated with COPD exacerbations) induces *PPP1R15A/GADD34* expression in epithelial cells likely involving activation of heme-regulated eIF2α kinase (HRI; [35]). In line with our observation on selective induction of GADD34 by NTHi, other studies also showed that microbial stimulation increases *PPP1R15A/GADD34* mRNA independent from *DDTI3/CHOP* mRNA induction [36, 37]. This may be relevant to COPD pathogenesis, since colonization by respiratory pathogens such as NTHi is a frequent finding in COPD patients [10] and may help to explain the presence of markers of activation of ISR as observed in COPD lung tissue [38].

Cellular exposure to diesel particles has been commonly associated with activation of an inflammatory response with an increase in markers such as CXCL8 and IL-6 [22, 39]. CXCL8 mediates recruitment of neutrophils, which are increased in the lung of COPD patients during an exacerbation [40]. We also observed that whole DE caused a moderate increase in *CXCL8* expression, which reached statistical significance when increasing power by merging the COPD and non-COPD cultures. However, the NTHi-induced *CXCL8* expression was not influenced by the previous DE exposure. Which constituents of the diesel mixture and which molecular pathways determine *CXCL8* induction is still unknown. For the first time, we showed that DE exposure limited the ability of the lung epithelium to respond to NTHi with an antimicrobial response in primary bronchial epithelial cells from COPD patients. Previously, *DEFB4A*/hBD2 mRNA was found to be reduced in submerged cultures of the alveolar cell line (A549) treated with diesel exhaust particles [11]. Furthermore, treatment with DEP impaired innate immune responses in primary peripheral blood mononuclear cell (PBMC) [13]. Our study adds to this information by showing that exposure of differentiated primary airway epithelial to whole DE, instead of aged DE particles in suspension, decreases NTHI-induced expression of *DEFB4A*/hBD2 and *S100A7*. Although this inhibition did not reach statistical significance due to substantial inter-donor variation, a DE-induced impairment of expression of these and other antimicrobial peptides may be highly relevant to COPD considering the strong link between both exposure to particulate air pollution and COPD exacerbations [7–9], and between NTHi and COPD exacerbations [10]. Three hours exposure to NTHi was insufficient to detect hBD-2 protein release. Therefore, further analyses of the antimicrobial response at the protein and functional level are required to elucidate the implications of our findings, and mechanistic studies are needed to delineate the underlying mechanisms. A previous study did suggest a mechanism by which PM_10_ and PM_2.5_ diesel particles reduce the *M. tuberculosis*-induced hBD2 expression in A549 epithelial cells, which was proposed to involve induction of cellular senescence [11]. However, such particles have been reported to also enhance the IL-1β-induced *DEFB4A* expression in the same A549 cells which was suggested to involve NF-kappaB signalling [41]. These apparently conflicting data do not provide a clear link to a mechanism. In addition, these previous studies were conducted using higher doses of aged diesel particles compared to the delivered dose of 0.40 μg/cm^2^ used in the present study. Furthermore, comparisons are limited by the different method of administration of diesel particles in a liquid-based delivery exposure system, and the use of the A549 tumor epithelial cell line, which do not mimic realistic exposure. No data were previously reported on the influence of diesel exhaust on S100A7 expression.

The use of air-liquid interface (ALI) cultures of primary bronchial epithelial cells (PBECs) provides several advantages for investigation of pulmonary defense mechanisms, and for toxicological studies involving exposure to complex mixtures such as diesel emissions. Furthermore, the ALI condition allows cells to differentiate, resembling the lung mucosa and at least partly maintaining the phenotypical characteristics of the disease state, as shown here by the differential expression of markers such as *MUC5AC* between COPD and controls donors. A limitation of the present study was that only cells from patients with mild-to-moderate COPD and not from those with more severe disease were available for this study, and no comparison to never-smokers was performed. Therefore putative differences in the response to DE may have been underestimated. Furthermore, although some responses appeared to be significant only in patients with COPD, we cannot formally exclude the possibility that the difference in size of the groups (*n* = 7 for COPD, and *n* = 5 for non-COPD) has contributed to this observation. Therefore, in addition to disease severity, also the small sample size of the two groups may help to explain the lack of statistical significant differences between the response of COPD donors and controls. A major advantage of using controlled exposure to whole diesel emissions (rather than resuspended particles) as performed in the present study, is the possibility to study exposure conditions relevant to real life exposure. In our previous study [21], we demonstrated the relevance of DE doses investigated in our *in vitro* model of exposure, where 1 h correspond to 2.25 h exposure *in vivo* to relatively high level of pollution. Such short periods of exposure did not lead to adverse effects on barrier and viability, suggests that chronic/repeated exposures to DE are feasible.

## Conclusion

In the present study we show that DE exposure causes activation of both an oxidative stress response and an integrated stress response in primary airway epithelial cells from both COPD patients and (ex)-smoking controls, without marked differences in their response. Furthermore, we showed that NTHi also causes selective activation of the ISR, which is further enhanced by prior DE exposure. Finally, we showed that DE exposure impaired the induction of *S100A7* expression by NTHi. These data suggest a potential link between diesel exposure and NTHi infection during COPD exacerbations, involving DE- and NTHI-induced activation of the ISR and DE-mediated alterations in the NTHI-induced innate immune response of the lung epithelium.

## Additional files

Additional file 1: Figure S1. MUC5AC and FOXJ1 basal expression in COPD and control donors. MUC5AC (oligomeric mucus/gel-forming, marker for mucus producing cells, 1A) and FOXJ1 (forkhead box J1, marker for ciliated cells, 1B) mRNA expression in untreated controls from COPD and control donors. Data are shown as normalized expression based on two reference genes, ATP5b and RPL13A. Statistical differences were studied with an independent nonparametric samples *t*-test. (PDF 71 kb)
Additional file 2: Figure S2. Tunicamycin-induced unfolded protein response. Cellular response of 5 non-COPD and 7 COPD donors treated for 3 h with 5 μg/ml tunicamycin (Tm) which was added to the basal compartment. DDIT3/CHOP (2A) and PPP1R15A/GADD34 (2B) mRNA expression is shown as fold from untreated controls after normalization on two reference genes, ATP5b and RPL13A. (PDF 275 kb)
Additional file 3: Figure S3. NTHi-induced inflammatory, antimicrobial response and HSPA5/BiP induction. Cellular response of 5 non-COPD and 7 COPD donors treated for 3 h with UV-NTHi added to the apical side. CXCL8 (3A), DEFB4A (3B), S100A7 (3C) and HSPA5/BiP (3D) mRNA expression is reported as fold from untreated controls after normalization on two reference genes, ATP5b and RPL13A. (PDF 492 kb)
Additional file 4: Figure S4. TNFα-induced inflammatory, antimicrobial response and HSPA5/BiP induction. Cellular response of cultures from 5 non-COPD and 7 COPD donors treated for 3 h with 20 ng/ml of TNFα added to the basal medium. CXCL8 (4A), DEFB4A (4B), S100A7 (4C) and HSPA5/BiP (4D) mRNA expression is reported as fold from untreated controls after normalization on two reference genes, ATP5b and RPL13A. (PDF 524 kb)

## Abbreviations

ALI: Air-liquid interface
ATF4: Activating transcription factor 4
ATF6: Activating transcription factor 6
ATP5b: ATP synthase subunit beta
BEBM: Bronchial epithelial growth media
BSA: Bovine serum albumin
COPD: Chronic obstructive pulmonary disease
CXCL8: Interleukin 8
DD: Delivered dose
*dd*: deposited dose
DDIT3/CHOP: DNA damage inducible transcript 3
DE: Diesel exhaust
DEFB4A/HBD2: Human defensin beta 4A
DMEM: Dulbecco’s Modified Eagle Medium
eIF2α: eukaryotic Initiation Factor 2
GCN2: General control nonderepressible kinase 2
HMOX1: Heme oxygenase-1 gene name
HO-1: Heme oxygenase-1 protein
HRI: Heme-regulated eIF2α kinase
HSPA5/BiP: Heat shock protein family A (Hsp70) member 5
IRE1α: Inositol-requiring enzyme 1
ISR: Integrated stress response
LCN2: Lipocalin-2
NTHi: Non-typeable *Haemophilus influenzae*
OD: Optical density
PBEC: Primary bronchial epithelia cells
PBS: Phosphate buffered saline
PERK: Protein kinase R (PKR)-like endoplasmic reticulum kinase
PKR: Protein kinase R
PM: Particulate matter
PPP1R15A/GADD34: Protein phosphatase 1 regulatory subunit 15A
RPL13a: Ribosomal protein L13A
S100A7: S100 calcium binding protein A7
SLPI: Secretory leukocyte protease inhibitor
SMPS: Scanning mobility particle sizer
splXBP1: spliced X-box binding protein 1
TGFβ: Transforming growth factor beta
TNFα: Tumor necrosis factor alpha
TSB: Tryptic Soy Broth
UPR: Unfolded protein response.
