# Supplementary figures and images for: Diesel exhaust alters the response of cultured primary bronchial epithelial cells from patients with chronic obstructive pulmonary disease (COPD) to non-typeable Haemophilus influenzae
Source: Respir Res. 2017 Jan 28;18:27. doi: 10.1186/s12931-017-0510-4 (PMC5273858; doi:10.1186/s12931-017-0510-4)

*MUC5AC*

A

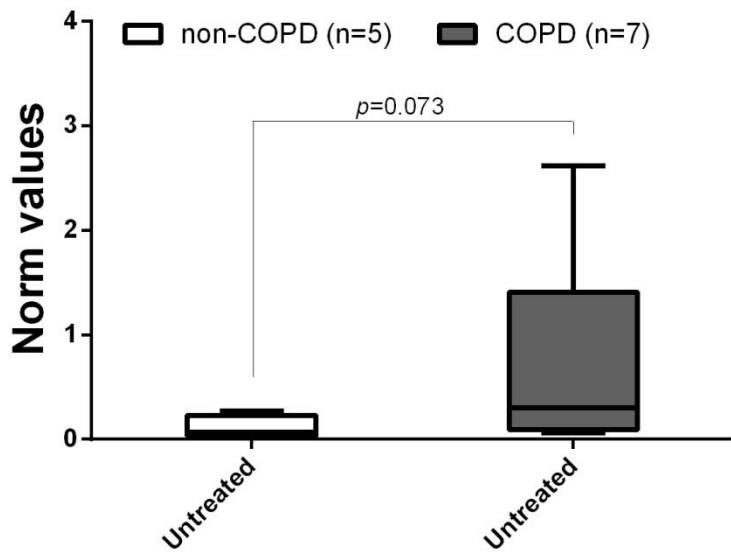

*FOXJ1*

B

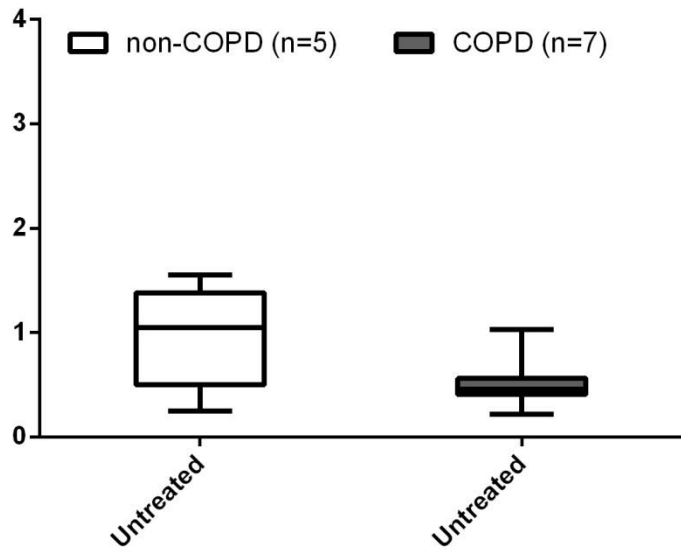

Supplement: Additional file 1: Figure S1. — MUC5AC and FOXJ1 basal expression in COPD and control donors. MUC5AC (oligomeric mucus/gel-forming, marker for mucus producing cells, 1A) and FOXJ1 (forkhead box J1, marker for ciliated cells, 1B) mRNA expression in untreated controls from COPD and control donors. Data are shown as normalized expression based on two reference genes, ATP5b and RPL13A. Statistical differences were studied with an independent nonparametric samples t-test. (PDF 71 kb) [file 12931_2017_510_MOESM1_ESM.pdf]

# **A** *DDIT3/CHOP*

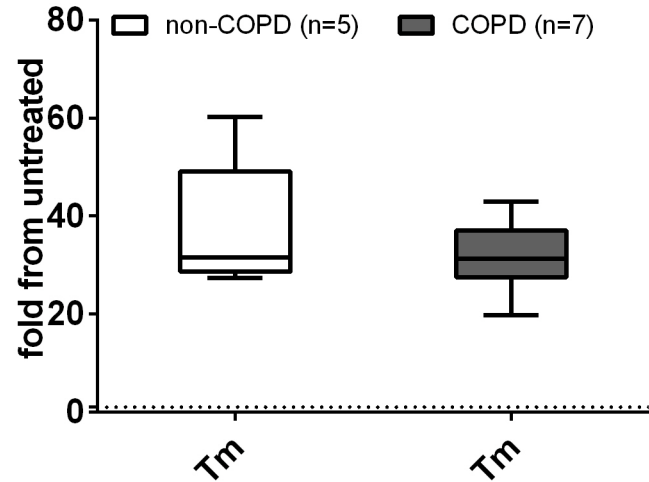

# **B** *PPP1R15A/GADD34*

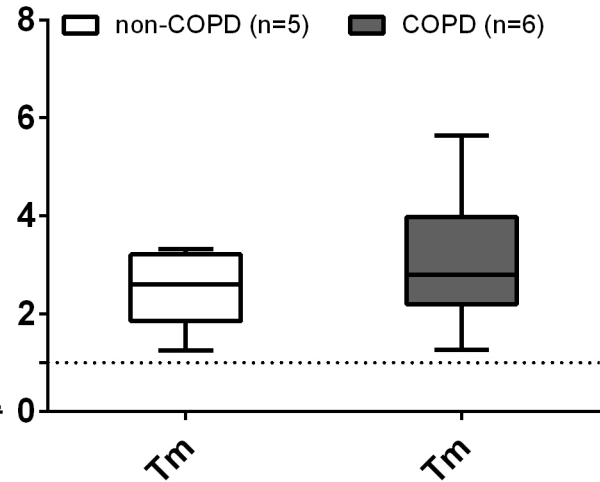

Supplement: Additional file 2: Figure S2. — Tunicamycin-induced unfolded protein response. Cellular response of 5 non-COPD and 7 COPD donors treated for 3 h with 5 μg/ml tunicamycin (Tm) which was added to the basal compartment. DDIT3/CHOP (2A) and PPP1R15A/GADD34 (2B) mRNA expression is shown as fold from untreated controls after normalization on two reference genes, ATP5b and RPL13A. (PDF 275 kb) [file 12931_2017_510_MOESM2_ESM.pdf]

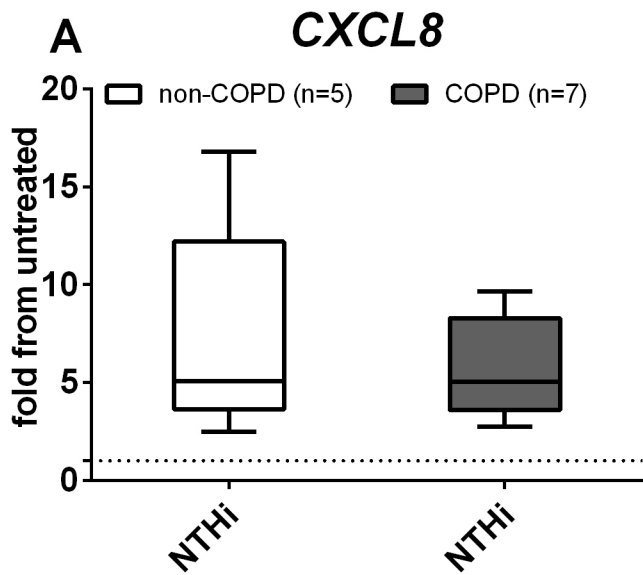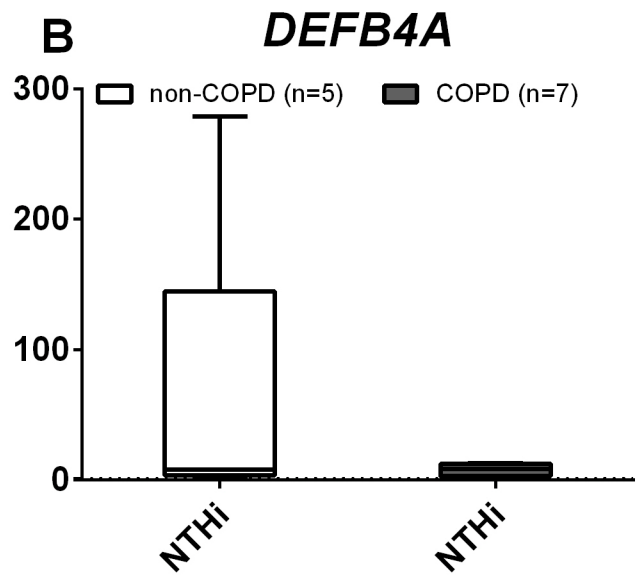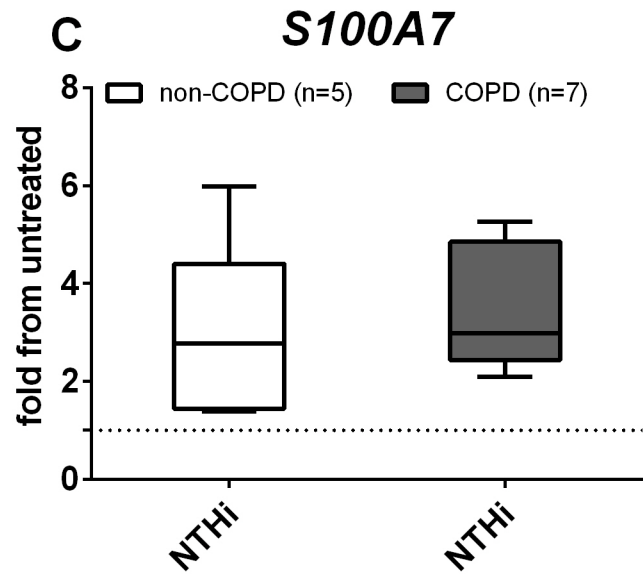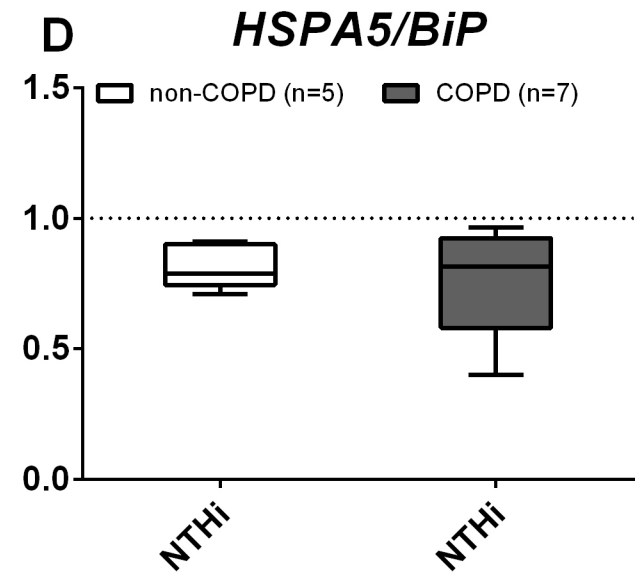

Supplement: Additional file 3: Figure S3. — NTHi-induced inflammatory, antimicrobial response and HSPA5/BiP induction. Cellular response of 5 non-COPD and 7 COPD donors treated for 3 h with UV-NTHi added to the apical side. CXCL8 (3A), DEFB4A (3B), S100A7 (3C) and HSPA5/BiP (3D) mRNA expression is reported as fold from untreated controls after normalization on two reference genes, ATP5b and RPL13A. (PDF 492 kb) [file 12931_2017_510_MOESM3_ESM.pdf]

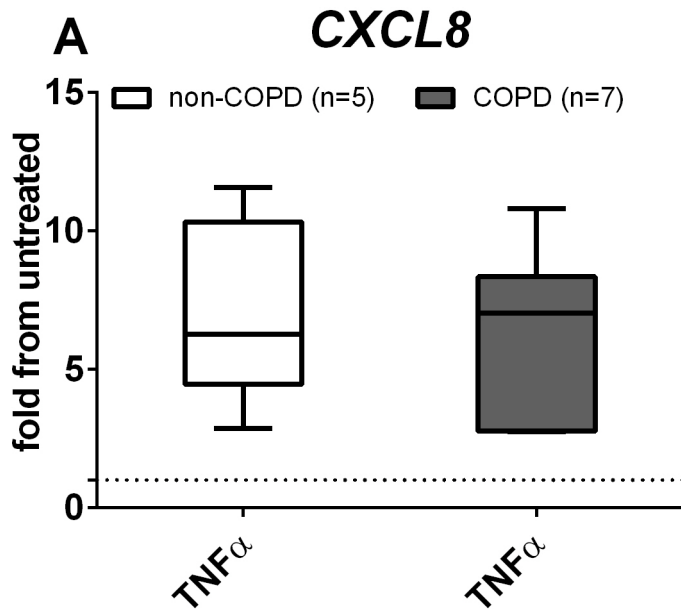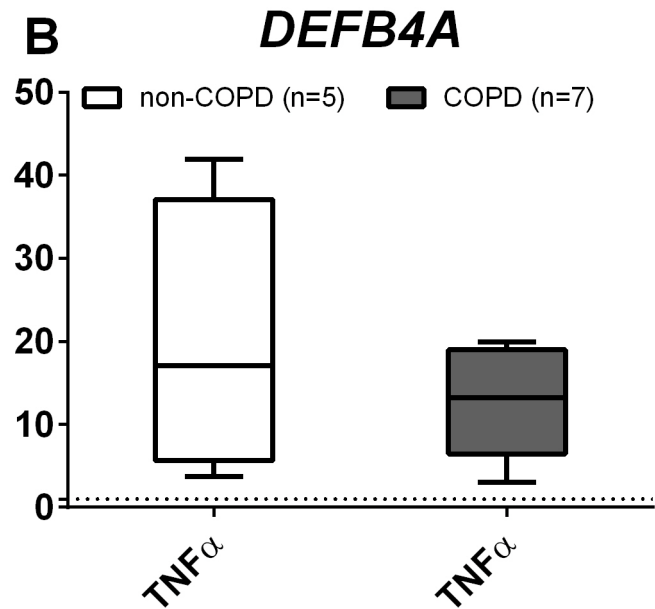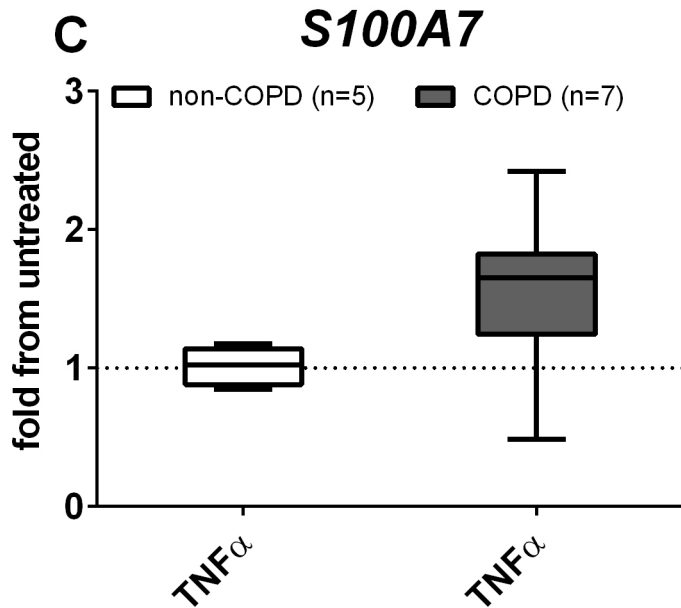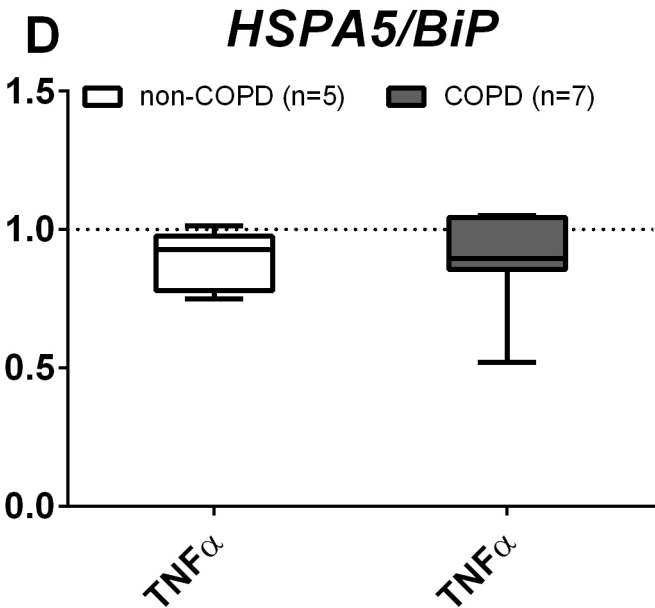

Supplement: Additional file 4: Figure S4. — TNFα-induced inflammatory, antimicrobial response and HSPA5/BiP induction. Cellular response of cultures from 5 non-COPD and 7 COPD donors treated for 3 h with 20 ng/ml of TNFα added to the basal medium. CXCL8 (4A), DEFB4A (4B), S100A7 (4C) and HSPA5/BiP (4D) mRNA expression is reported as fold from untreated controls after normalization on two reference genes, ATP5b and RPL13A. (PDF 524 kb) [file 12931_2017_510_MOESM4_ESM.pdf]
